# Characterizing Growth-Retarded Japanese Eels (*Anguilla japonica*): Insights into Metabolic and Appetite Regulation

**DOI:** 10.3390/metabo14080432

**Published:** 2024-08-05

**Authors:** Xiangbiao Zeng, Jingwei Liu, Yiwen Chen, Huan Han, Yanhe Liu, Bin Xie, Tianwei Jiang, Chris Kong-Chu Wong, Kang Li, Liping Liu

**Affiliations:** 1 China-ASEAN Belt and Road Joint Laboratory on Mariculture Technology (Shanghai), Shanghai Ocean University, Shanghai 201306, China; xiangbiaoz_shou@163.com (X.Z.); jackcyw@126.com (Y.C.); tom220100209@st.shou.edu.cn (H.H.); cristbradford2@gmail.com (Y.L.); 15575135437@163.com (B.X.); 13770451858@163.com (T.J.); kli@shou.edu.cn (K.L.); 2 Key Laboratory of Exploration and Utilization of Aquatic Genetic Resources, Ministry of Education, Shanghai Ocean University, Shanghai 201306, China; 3 Shanghai Engineering Research Center of Aquaculture, Shanghai Ocean University, Shanghai 201306, China; 4 Department of Biology, Croucher Institute for Environmental Sciences, Hong Kong Baptist University, Hong Kong SAR, China; ckcwong@hkbu.edu.hk

**Keywords:** Japanese eel, growth differences, size uniformity, appetite-related genes, metabolism

## Abstract

During field surveys and culture procedures, large growth disparities in *Anguilla japonica* have been observed. However, the potential causes are unknown. This study explored differences in digestive ability, metabolic levels, and transcriptomic profiles of appetite-related genes between growth-retarded eel (GRE) and normal-growing eel (NGE) under the same rearing conditions. The results showed that growth hormone (*gh*) mRNA expression in GREs was considerably lower than NGEs. The levels of total protein (TP), total cholesterol (T-CHO), triglyceride (TG), low-density lipoprotein cholesterol (LDL-C), high-density lipoprotein cholesterol (HDL-C), blood ammonia (BA), blood urea nitrogen (BUN), and alkaline phosphatase (ALP) in GREs were significantly lower than in NGEs. Conversely, levels of glucose (GLU), alanine aminotransferase (ALT), and aspartate transaminase (AST) were higher in GREs. The activities of SOD, CAT, and T-AOC levels were also significantly lower in GREs, as were the activities of glucose-related enzymes including hexokinase (HK), pyruvate kinase (PK), phosphoenolpyruvate carboxykinase (PEPCK), and glucose-6-phosphatase (G6PASE). Additionally, orexigenic genes (*npy* and *ghrelin*) were dramatically downregulated, whereas anorexigenic genes (*crh* and *pyy*) were significantly upregulated in GREs. These findings suggested that variances in growth hormone, metabolic activities, and appetite level could be associated with the different growth rates of *A. japonica*. The present research not only revealed the characteristics of the growth, metabolism, and appetite of GREs but also offered new perspectives into the substantial growth discrepancies in *A. japonica*, providing novel ideas for enhancing fish growth.

## 1. Introduction

Growth rate and size uniformity are important indicators in fish culture, as they have a significant impact on economic benefit and culture management [1]. However, many fish species show considerable intra-population size disparities during the growth phase. These species include giant grouper (*Epinephelus lanceolatus*) [2], spotted seabass (*Lateolabrax maculatus*) [3], black porgy (*Acanthopagrus schlegelii*) [4], nile tilapia (*Oreochromis niloticus*) [5], and swamp eels (*Monopterus albus*) [6].

Numerous studies have been conducted to uncover the riddle of growth rate variability. But growth is a complicated process that is influenced by a variety of internal and external factors. Genetic factors, hormone levels, social hierarchy, physiological factors, and environmental conditions have all been linked to fish growth performance [7,8]. Among these hypotheses, differences in growth-related hormone levels [9,10] and metabolic performance [5,11] are two of the main reasons for the discrepancy in growth. The growth hormone (GH)-insulin-like growth factor (IGF) system serves as the primary regulatory mechanism governing the growth of vertebrates [12,13]. Furthermore, ample research has demonstrated a profound link between metabolism and growth in fish. For instance, investigations on rainbow trout (*Oncorhynchus mykiss*) have revealed that individuals exhibiting a faster growth rate possess a heightened metabolic capacity [11]. Similarly, studies on fast-growing tuna have shown enhanced metabolic capabilities, suggesting a correlation between growth rate and intrinsic metabolic potential [14].

It is widely acknowledged that vital nutrients and energy are necessary for somatic growth [15]. These energy sources are derived from food and subsequently converted into cellular and tissue components through metabolic processes. Consequently, food intake and the regulation of energy balance play pivotal roles in the intricate growth regulatory network of vertebrates, alongside the sensing of growth hormone and the metabolic status [16,17]. The appetite system, which governs food intake, is also closely linked to fish growth and metabolic activities [18]. The regulation of appetite in fish is a complex interplay between the central nervous system, particularly the hypothalamus, and peripheral signals originating from neurosensory inputs, blood-borne nutrients, and hormones [17]. The hypothalamus contains two distinct neuronal clusters that integrate diverse cerebral inputs and peripheral signals: the orexigenic center, which promotes food intake, and the anorexigenic center, which inhibits it. Key orexigenic factors include neuropeptide Y (*npy*) and *ghrelin*, while anorexigenic factors comprise corticotropin-releasing hormone (*crh*), pro-opiomelanocortin (*pomc*), cholecystokinin (*cck*), and peptide YY (*pyy)* [17]. This intricate interplay between the central and peripheral signals ensures the precise regulation of appetite, thereby influencing fish growth and metabolic functions.

The Japanese eel (*Anguilla japonica*) is a commercially important fish with a high nutritional value and market price [19,20]. However, the resources of wild *A. japonica* have been depleted due to environmental pollution and overfishing [21]. Under captive rearing conditions, the growth rate of eel exhibits considerable variability [22]. A previous study discovered that after 60 days of rearing Pacific shortfin eel (*Anguilla bicolor pacifica*) with similar starting weights, the coefficient of variation escalated to a staggering 34.14% [23]. Indeed, growth-retarded eels (GREs) are generally characterized by their small size, reduced food consumption, and extremely slow growth rates [24]. These GREs constitute approximately 10–20% of the total population, resulting in substantial financial losses. Several factors, including inadequate starter feeding, detrimental environmental conditions, and disease infections have been suggested as potential contributors to the occurrence of GREs [25].

Previous research efforts on GREs have primarily concentrated on strategies to promote growth, such as incorporating anthocyanins and plant extracts into their feed [24,25,26]. While these interventions have shown some positive effects on growth performance, they have not consistently achieved satisfactory outcomes. To date, the underlying causes of the significant growth variability among eels remain uncertain. We hypothesized that appetite regulation may play an important role in the growth discrepancies in *A*. *japonica* and the formation of the GREs. To test the hypothesis, in the present study, we utilized *A. japonica* of similar ages but with substantial growth differences under identical culture conditions as the experiment models of GREs and normal-growing eels (NGEs). We conducted a comparative analysis of the blood biochemistry, metabolic enzymes, and the expression of growth- and appetite-related genes between GREs and NGEs. The findings may provide a novel perspective for enhancing the control of the growth rates and achieving greater size uniformity in *A. japonica* culture practice.

## 2. Materials and Methods

### 2.1. Ethics Statement

The handling and culture of the animals used in this research study were carried out in compliance with the guidelines established by the Animal Ethics Committee of Shanghai Ocean University (Shanghai, China), following the approved protocol numbers SHOU-DW-2020-017 and SHOU-DW-2023-070.

### 2.2. Fish Maintenance and Samples Collection

The experiment was carried out at the Chongming Aquaculture Cooperative of Shanghai Ocean University. In July 2022, 6000 healthy *A. japonica* elvers of similar size (5.21 ± 0.06 g) were purchased and transferred to the circulating water system. The aquaculture system comprised three tanks, each with a diameter of 6 meters and a depth of 1 meter, equipped with continuous aeration. The commercial powder diet was mixed with water at a ratio of 1:2 to form dough, which was fed to the fish twice daily (at 08 h00 and 18 h00). The diet had the following approximate compositions: moisture (<10%), crude protein (>47%), crude fat (>4%), lysine (>2.5%), crude fiber (<3%), ash content (<17%), and total phosphorus (1% to 2.8%). The daily feeding rate was set at 2% to 3% of the body weight. Dissolved oxygen in the water was kept above 6 mg·L^−1^, the ammonia nitrogen level was kept below 0.3mg·L^−1^, and water temperature was maintained between 25 ± 2 °C. About 12 h of natural daylight were provided and 20% of the water was changed daily.

In July 2023, after a year of culturing, the weight and length of the eels from the three aquatic tanks were measured. The 30 eels were randomly sampled from each tank, with a total of 90 eels used to calculate the average weight (380 ± 57.66 g). Eels with weight close to the average weight were designated as “normal-growing eel (NGE)”, while those exhibiting significantly slower growth were classified as “growth-retarded eel (GRE)”.

The 30 NGEs and 30 GREs were selected, anesthetized (100 mg·L^−1^ tricaine methane sulfonate; MS222; Sigma-Aldrich, St. Louis, MO, USA), and individually weighed for growth performance evaluation. In each group, 9 eels were chosen for blood biochemical, enzymatic activity, and qRT-PCR analyses. Three samples were formed by mixing of 3 fish and replicated 3 times. Blood was obtained by puncturing the caudal vein using a 1 mL syringe and kept on ice for two hours for serum separation, followed by centrifugation at 2664× *g*/10 min at 4 °C. The supernatant was collected and stored at −80 °C until further biochemical analyses. Subsequently, the fish were dissected, and their brains, stomachs, intestines, and livers were collected. Additionally, 3 fish from each group had 0.5 cm segments of their intestines preserved in 4% paraformaldehyde for histological examination. Specific details of the experimental design are shown in Figure S1.

### 2.3. Growth Parameters Analysis

After 24 h of feed deprivation, the growth performance of 30 NGEs and 30 GREs was calculated. Hepatosomatic index (HSI), viscerosomatic index (VSI), condition factor (CF), specific growth rate (SGR), and weight gain rate (WGR) were calculated as follows:

HSI (%) = liver weight/body weight × 100.

VSI (%) = visceral mass weight/body weight × 100.

CF (g/cm^3^) = body weight/body length^3^.

SGR (%) = (ln*W_t_* − ln*W*_0_)/days × 100.

WGR (%) = (*W_t_* − *W*_0_)/*W*_0_ × 100.

*W_t_* is the body weight of eel after one year of culture; *W*_0_ is the initial body weight.

### 2.4. Blood Biochemical Analysis

The activities of total proteins (TP, No. A045-4-2), glucose (GLU, No. A154-1-1), albumin (ALB, No. A028-2-1), alkaline phosphatase (ALP, No. A059-2-2), blood urea nitrogen (BUN, No. C013-1-1), alanine aminotransferase (ALT, No. C009-2-1), blood ammonia (BA, No. A086-1-1), aspartate aminotransferase (AST, No. C010-2-1), high-density lipoprotein cholesterol (HDL-C, No. A112-1-1), low-density lipoprotein cholesterol (LDL-C, No. A113-1-1), total cholesterol (T-CHO, No. A111-1-1), and triglycerides (TG, No. A1100-1-1) were determined using a microplate scanning spectrophotometer (Thermo scientific, New York, NY, USA) and commercially available kits (Nanjing Jiancheng Bioengineering Institute, Nanjing, China). The wavelengths for measuring TP, GLU, ALB, ALP, BUN, ALT, BA, AST, HDL-C, LDL-C, T-CHO, and TG are 595, 505, 628, 520, 640, 505, 630, 510, 600, 600, 500, and 500 nm, respectively. All experimental procedures were conducted following the manufacturer’s protocols.

### 2.5. Analysis of Digestive Enzymes in Intestine and Antioxidant, Metabolic Enzyme in Liver

The intestines and livers tissues were cleaned with ice-cold sterilized normal saline (0.9% sodium chloride), and weighed. The samples were homogenized in ice-cold sterilized normal saline (weight: volume ratio = 1: 4 or 1:9 g·mL^−1^) with an OSE-Y50 tissue grinder (Tiangen Biotech Co., Ltd., Beijing, China) and centrifuged for 10 min at 1998× *g*. The supernatant was collected and stored at −20 °C for subsequent examination. Enzyme activities were measured using diagnostic reagent kits from Nanjing Jiancheng Bioengineering Institute (Nanjing, China) and experimental procedures were conducted in accordance with their protocols. The digestive enzymes (lipase, No. A054-1-1; amylase, No. C016-2-1 and trypsin, No. A080-2-1), antioxidant enzymes (superoxide dismutase, SOD, No. A001-1; catalase, CAT, No. A007-2-1; total antioxidant capacity, T-AOC, No. A015-3-1 and malondialdehyde, MDA, No. A003-1), and metabolic enzymes (hexokinase, HK, No. A077-3-1; pyruvate kinase, PK, No. A076-1-1; lactic dehydrogenase, LDH, No. A020-2-1; phosphoenolpyruvate carboxykinase, PEPCK, No. A131-1-1; glucose-6-phosphatase, G6PASE, H580-1-1) were determined. Among these indexes, SOD (450 nm), CAT (405 nm), T-AOC (405 nm), MDA (532 nm), LDH (440 nm) were determined using a microplate scanning spectrophotometer. The lipase (580 nm), amylase (660 nm), trypsin (253 nm), HK (340 nm), PK (340 nm), PEPCK (340 nm), and G6PASE (450 nm) were determined by a spectrophotometer (UV5100, METASH, Shanghai, China).

### 2.6. Histomorphology

About 0.5 cm mid-intestine was sampled from 3 NGEs and 3 GREs and fixed with 4% paraformaldehyde. The samples were then fixed, dehydrated, embedded, and sectioned (4–5 μm) with a Leica RM2016 Microtomes paraffin slicer (Leica, Weztlar, Germany). Paraffin slices were deparaffinized and rehydrated before being stained with hematoxylin-eosin (HE) solution. The stained sections were sealed with neutral glue and examined under a light microscope (Eclipse, Ni-E, Nikon; Tokyo, Japan). Goblet cells were quantified on each intestinal villus, and statistical analysis was conducted using the average value per sample as the measurement data.

### 2.7. Extraction of RNA, cDNA Synthesis, Primer Design and qRTPCR

RNase-free steel beads were added into samples (brain, intestine, and stomach) and homogenized at 70 Hz for 4 min. Total RNA was extracted using TRI reagent (Sigma-Aldrich, MO, USA). The NanoDropND-2000C (Thermo, Waltham, MA, USA) was used to determine total RNA concentration and purity. Evo M-MLV Reverse Transcription Premix Kit (Accurate Biology, Changsha, China) was used to reverse-transcribe 1 μg of RNA into cDNA.

Through NCBI, the target gene’s entry number was located, and the CDS sequence was acquired. Prime 6 software was utilized to design targeted primers, with *EF1α* as the internal reference gene (GenBank accession number EU407824) (Table S1). A linear regression model was used to generate standard curves for each primer from a 10-fold serial dilution of cDNA. Amplification was performed in triplicate on a Bio-Rad CFX96 (Bio-Rad, Hercules, CA, USA) with a SYBR^®^ Green Premix Pro Taq HS qPCR Kit from Accurate Biology in Changsha, China. Each 20 μL reaction included 10 μL of 2× SYBR^®^ Green Pro Taq HS Premix II, 0.8 μL of forward primer (10 mol/L), 0.8 μL of reverse primer (10 mol/L), 6.8 μL of ddH2O, and 1.6 μL of cDNA (equivalent to 100 ng of total RNA). Additionally, for each primer combination, non-template controls were also supplied. Forty cycles of 95 °C for 3 min, 95 °C for 5 s, and 60 °C for 30 s were used for the amplification process. Throughout the extension phase, signals were recorded. Before the fluorescence signal of the dissolution curve was recorded, the temperature was raised by 0.5 °C for 5 s on each cycle, from 65 to 95 °C. For the RT-PCR analyses, three technical and three biological duplicates were used. The 2^−∆∆CT^ method was used to analyze the amplification results and assess the expression level of each sample in relation to the internal reference gene *EF1α*.

### 2.8. Statistical Analysis

SPSS 26.0 software was used for Levene’s Test for Equality of Variances and Independent Samples t-test for growth traits, blood biochemical, enzyme activities, and RT-PCR comparison between NGEs and GREs. The significance level was * *p* < 0.05. *p*-values < 0.01 and *p*-values < 0.001 are reported as ** *p* < 0.01 and *** *p* < 0.001, respectively. All data are presented as mean ± SD.

## 3. Results

### 3.1. Growth Performance and Growth-Related Genes Expression

**Figure 1:**
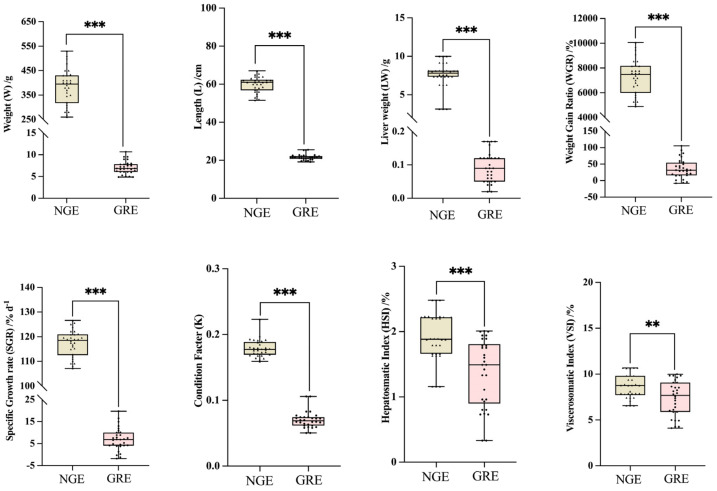
Growth traits and analysis of significant differences between normal-growing eel (NGE) and growth-retarded eel (GRE). Each black triangle represents an NGE individual, while each black dot represents a GRE individual. Significant differences between the two groups are identified with different markers (** *p* < 0.01; *** *p* < 0.001). All data are presented as mean ± SD (*n* = 30).

**Figure 2:**
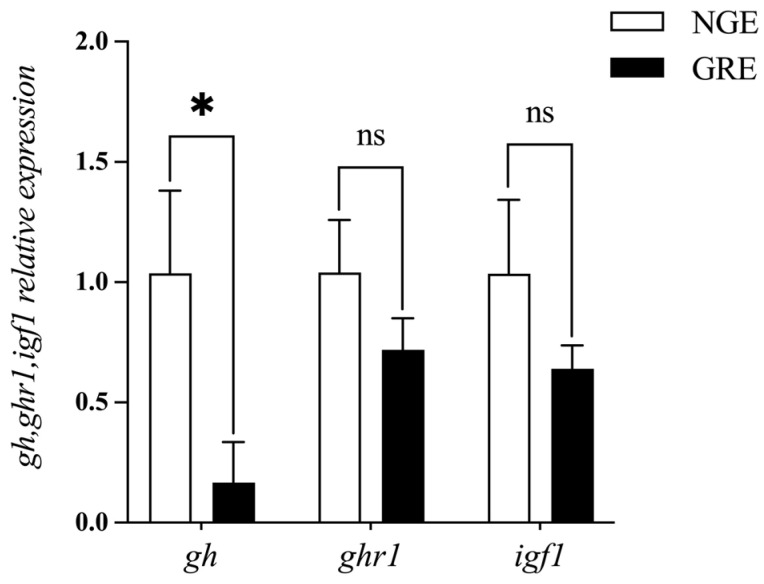
Comparison of the mRNA expression of brains’ growth hormone (*gh*), growth hormone receptor 1 (*ghr1*), and insulin-like growth factors (*igf1*) in NGE and GRE. Significant differences between the two groups are identified with different markers (* *p* < 0.05; ns, no significant difference).

Compared to the NGEs, the GREs exhibited significantly lower body weight, total length, WGR, SGR, CF, HSI, and VSI (*p* < 0.01, Figure 1). There was no statistically significant difference in the relative mRNA levels of *ghr1* and *igf1* between two groups (*p* > 0.05). However, the mRNA level of *gh* was N-fold lower in GRE compared to NGE (*p* < 0.05, Figure 2).

### 3.2. Serum Parameters

Significantly lower levels of TP, BA, BUN, T-CHO, TG, HDL-C, LDL-C, and ALP were revealed in GREs compared to NGEs (*p* < 0.05). Conversely, the levels of GLU, AST, and ALT were significantly higher in GREs (*p* < 0.05). No significant difference was found in serum ALB levels between the two groups (*p* > 0.05, see Table 1).

### 3.3. Antioxidant Enzyme Activities

**Figure 3:**
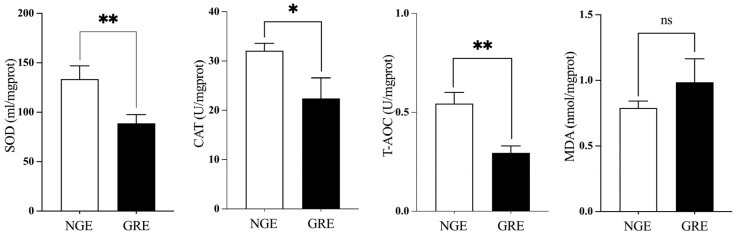
Comparative analysis of liver antioxidant capacity (superoxide dismutase, SOD; catalase, CAT; total antioxi-dant capacity, T-AOC and malondialdehyde, MDA) between NGE and GRE. Significant differences between the two groups are identified with different markers (* *p* < 0.05; ** *p* < 0.01; ns, no significant difference).

The results demonstrated that GREs exhibited significantly lower enzymatic activities of SOD, CAT, and T-AOC in the liver compared to NGEs (*p* < 0.05). Nonetheless, MDA levels were similiar between the GREs and the NGEs (*p* > 0.05, Figure 3).

### 3.4. Digestive Enzyme Activities and Intestinal Histology

Compared to NGEs, the enzymatic activities levels of lipase, trypsin, and amylase were suppressed in GREs (*p* < 0.05, Supplemental Figure S2). Additionally, *amylase* mRNA levels were significantly lower in the liver of GREs compared to the NGEs (*p* < 0.05). However, the levels of *lipase* and *trypsin* mRNA were no significant difference (*p* > 0.05, Figure S2).

The cross-section of intestinal tissue (Figure S3) revealed reduced intraepithelial goblet cells in the mid-intestinal mucosa of GREs (7.83 ± 1.57) compared to NGEs (28.83 ± 7.43). Additionally, the connection between the mid-intestinal submucosa and the muscle layer appeared looser in GREs.

### 3.5. Metabolic Enzyme Activities

**Figure 4:**
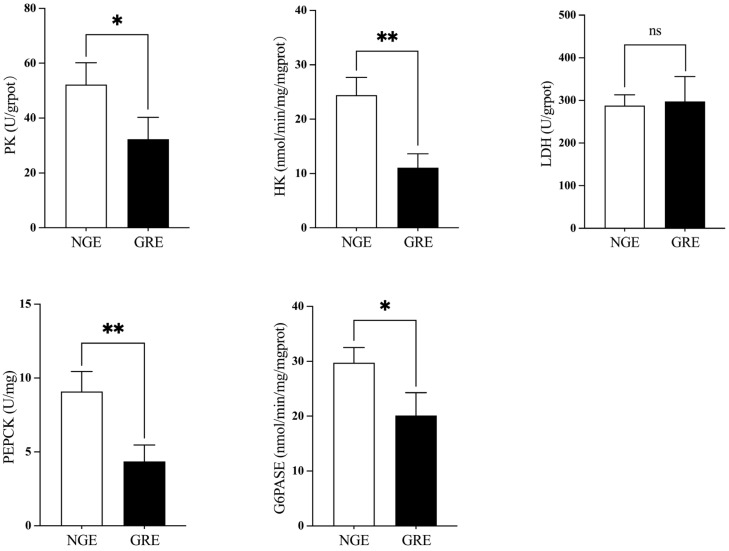
Comparative analysis of liver glucose metabolism enzymes (hexokinase, HK; pyruvate kinase, PK; lactic dehydrogen-ase, LDH; phosphoenolpyruvate carboxykinase, PEPCK; glucose-6-phosphatase, G6PASE) between NGE and GRE. Significant differences between the two groups are identified with different markers (* *p* < 0.05; ** *p* < 0.01; ns, no significant difference).

The activities of HK and PEPCK, PK and G6PASE were significantly higher in NGEs compared to GREs (*p* < 0.05, Figure 4). However, no significant difference in LDH activity was found between NGEs and GREs (*p* > 0.05).

### 3.6. Appetite-Related Genes Expression

**Figure 5:**
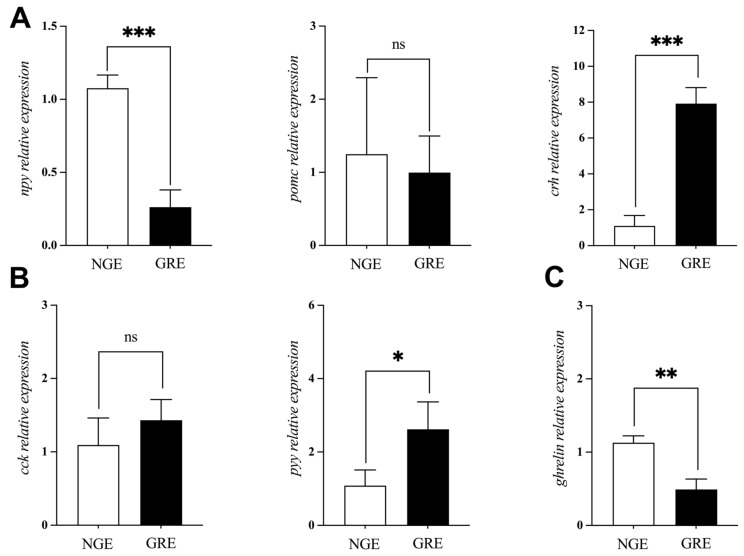
Analysis of appetite-related genes expression difference between NGE and GRE mRNA of (**A**) neuropeptide Y (*npy*), pro-opiomelanocortin (*pomc*) and corticotro-pin-releasing hormone (*crh*) in brain; (**B**) cholecystokinin (*cck*) and peptide YY (*pyy*) in intestine; and (**C**) *ghrelin* in stomach. Significant differences between the two groups are identified with different markers (* *p* < 0.05; ** *p* < 0.01; *** *p* < 0.001; ns, no significant difference).

The relative mRNA level of appetite-related genes was shown in Figure 5. The t-test indicated significantly lower relative expression of *npy* and *ghrelin*, whereas *crh* and *pyy* were significantly higher in GREs compared to NGEs (*p* < 0.05). No significant difference was observed in the mRNA level of *pomc* and *cck* genes between NGEs and GREs (*p* > 0.05).

## 4. Discussion

Fish growth performance is affected by various factors: species, genetics, environmental conditions, feeding patterns, nutrition, etc. It is not uncommon to observe significant variation in growth rates among individuals of the same species under identical rearing conditions. Such discrepancies can result in uneven fish sizes, thereby increasing market management costs. Previous studies have shown that the growth differences could be due to the variations in genetic background, hormone secretion, digestion, and metabolism levels. However, the most crucial mechanisms underlying fish growth remain not fully understood. The aim of this study was to explore the potential mechanisms contributing to severe growth retardation in *A. japonica* by examining the growth, digestion, metabolism, and appetite levels in GREs.

In the present study, *A. japonica* exhibited significant large growth disparities within a year, consistent with previous studies [27,28] and field investigations. Notably, in eel culture, not only will some eels have slower growth, but also a few individuals may even experience negative growth. The regulation of somatic growth in fish growth traits is mostly dependent on the GH-GHR-IGFs neuroendocrine pathway along the hypothalamic–pituitary growth axis [29]. GH is a pleiotropic hormone that regulates various biological functions, including development and growth [30]. Its growth-promoting effects have been widely demonstrated [31]. Our results showed that GREs had a significantly lower mRNA level of *gh* in the brain compared to NGEs. The inhibited *gh* expression in the brain could contribute to the slow growth observed in GREs. Supporting this hypothesis, a study reported that continuous oral recombinant gh feeding to *A. japonica* larvae resulted in a considerable increase in eel body size [32], confirming the growth-promoting effect of gh. In addition, slow-growing *O. niloticus* exhibited lower *gh* levels in the pituitary compared to their fast-growing counterparts [33].

Serum biochemical analysis is a reliable indicator of metabolic and bodily health issues [34]. The significant decrease in TP levels and altered lipid profiles (T-CHO, TG, HDL-C, LDL-C) in the blood of GREs compared to NGEs suggests impaired liver function, reduced protein metabolism, and lower lipid metabolism in GREs [35]. This finding is consistent with results observed in *O. niloticus* [36]. The urea nitrogen and ammonia concentration of serum is an essential indicator for assessing protein synthesis in fish [37]. The GRE’s low blood ammonia and urea nitrogen levels indicate a reduced level of amino acid metabolism. Similarly, *A. schlegelii* has been reported to exhibit poor protein synthesis and utilization ability in individuals with low growth performance [4]. As a metabolic regulating enzyme, ALP plays a critical role in the nonspecific immune response, inflammation modulation, and enhancement of immune cell communication and activity in fish [38]. Elevated ALP levels in NGEs indicate a stronger innate immune system, benefiting fish health and resistance to infections, and may accelerate their growth rate. It is noteworthy that NGEs showed lower plasma glucose level than GREs. This phenomenon was also discovered in a study on *O. niloticus* [36]. This may indicate an excess glucose consumption in fast-growing fish thus leading to a lower glycemia level when compared to the growth-retarded individuals. Further research into the specific regulatory process is required.

Glycolysis and gluconeogenesis both are major pathways of glucose metabolism. According to Li et al. [39], glycolytic-related genes (*hk*, *gapdfh*, *pk*, *ldh*, *gpi*) were all elevated in fast-growing *A. dabryanus*, showing that the glycolytic process was increased and favorably connected with growth performance. This is consistent with the findings of this study, which suggest that the energy utilization of carbohydrates in eels with high growth performance may be greater, potentially playing a crucial role in enhancing growth performance. Similar findings were seen in *Larimichthys crocea*, where a significant number of DEGs between individuals with different growth rates were categorized as a glycolytic route, and glycolysis was discovered to be related with individual growth [40]. The primary enzymes involved in gluconeogenesis are PEPCK and G6PASE [41]. Fast-growing *O. niloticus* exhibited substantially higher hepatic G6PASE expression compared to fish with low growth performance [5], which is consistent with our findings. However, the expression of key genes involved in gluconeogenesis was higher in slow-growing sea cucumbers. This could be related to slow-growing sea cucumbers’ lower food intake and hence require more glucose from the gluconeogenic pathway to maintain life-sustaining activities [42]. 

The antioxidant capacity of fish can serve as an indicator of their health status. Antioxidant responses are typically generated under stressful conditions to mitigate negative effects on fish [43,44]. MDA, a harmful byproduct of lipid peroxidation, is used to measure the extent of oxidative damage in the body. CAT, SOD, and T-AOC are commonly employed to assess the antioxidant capacity and health of the body. These biomarkers effectively neutralize reactive oxygen species, reduce oxidative damage, and enhance immunity [45,46]. The results in this study revealed that NGE have a higher antioxidant capacity. Similar results were reported in Yangtze sturgeon (*Acipenser dabryanus*) [47]. 

Multiple neurons and peripheral inputs collaborate intimately to regulate appetite, integrating these signals within the hypothalamus through dedicated systems that oversee hunger and satiety sensations [48]. In *A. japonica*, several appetite-modulating factors have been pinpointed, such as *ghrelin*, *pomc*, *crh*, *cck*, *pyy*, and *npy* [49,50,51,52]. In fish, one of the strongest orexigenic signals is *npy*, which is extensively expressed in both peripheral and central neural networks [53]. Food deprivation triggers an upsurge in *npy* expression in the brains of various fish species, including salmon (*Salmo salar*) [54] and goldfish (*Carassius auratus*) [55]. Results from the present study revealed a marked decrease in *npy* expression in the brains of GREs compared to NGEs, hinting at compromised appetite in GREs. This finding aligns with similar observations made in bighead carp, where *npy* expression levels were found to be higher in the intestines and hypothalamus of larger individuals compared to small ones [56]. Additionally, other studies have demonstrated a positive association between *npy* expression and growth hormone secretion [53,57,58,59], reinforcing the notion that *npy* plays a pivotal role in regulating growth-related processes, which is consistent with our current findings. On the other hand, *ghrelin* is primarily generated in fish stomachs and is involved in intestinal motility, growth hormone release, feed intake, and energy homeostasis [60]. *Ghrelin* has been demonstrated to have orexigenic effects on some fish species, including *O. niloticus* [61], grass carp (*Ctenopharyngodon idellus*) [62], brown trout (*Salmo trutta*) [63], and *C. auratus* [64]. GREs have a reduced appetite, as indicated by the substantially lower *ghrelin* expression in their stomach compared to NGEs. In contrast to *npy*, existing data demonstrate that *crh* and *pyy* have an anorexic role [48]. It has been reported that crh injection or ingestion can dramatically lower *C. auratus* food intake [65], while the expression level of *crh* in the brain of Ya fish (*Schizothorax prenanti*) under fasting conditions is significantly reduced [66]. *Pyy* is a peptide produced in the distal intestine that weakens appetite in mammals by inhibiting *npy* and activating *pomc* neurons [67]. Its anorexigenic effects have been observed in piranha (*Pygocentrusnattereri*) [68] and *C. idellus* [69]. Consequently, the higher *crh* and *pyy* level in the GREs may reflect its negative urge for food. 

Together, the findings of this experiment suggested that eels exhibiting low growth performance have suppressed appetite, offering insight into the potential factors contributing to the formation of slow-growing individuals. This raises an intriguing question: What are the underlying reasons for the reduced appetite and metabolic rate in GREs? We hypothesize that GRE may be attributed to adaptation to social hierarchy and environmental conditions, primarily manifesting as self-protective behavior. Eels experience significant inter-individual competition. For timid, less robust, or lower-status individuals, insufficient access to adequate food over extended periods may lead them to actively reduce appetite and metabolic rates to minimize energy expenditure, thereby enhancing their survival prospects. Further experiments are needed to validate this hypothesis. Furthermore, the growth rate of fish can also be influenced by various behavioral aspects, including feeding patterns, social hierarchy, aggression, stress levels, and the aggregating or isolating behavior. Fish displaying aggressive feeding behaviors, occupying a dominant position within their social hierarchy and experiencing reduced stress levels, are often observed to have faster growth rates [7]. Consequently, beyond the physiological considerations addressed in this study, enhancing our understanding and effective management of behavioral factors offers a promising avenue to promote more consistent and optimal growth among fish in aquaculture settings.

## 5. Conclusions

In this study, we investigated changes in blood biochemistry, digestive and metabolic enzyme activities, and gene expression patterns associated with growth and appetite in *A. japonica*, a species exhibiting notable growth variations despite being reared under the same culture conditions. We found reduced lipid, amino acid, and glucose metabolism in GREs compared to NGEs. In addition, GREs exhibited decreased *gh* mRNA expression and altered expression of appetite-related genes. These findings collectively point towards metabolic shifts and transcriptional modifications of *gh* and appetite-related genes as potential contributors to the observed growth disparities among *A. japonica*. Moreover, we speculate that the growth disparity among eels could be mitigated through measures such as (1) decreasing inter-individual competition, (2) enhancing appetite and metabolic rates, and (3) improving rearing conditions. This study offers new insights into the metabolic and appetite differences between *A. japonica* with considerable growth disparities under the same rearing conditions. It establishes a robust scientific basis for enhancing fish growth performance and underscores the importance of further research.

## Figures and Tables

**Table 1:** Comparative analysis of blood biochemical indexes of NGE and GRE.

	NGE	GRE	NGE vs. GRE
TP (g/L)	71.52 ± 1.58	55.60 ± 8.67	**
ALB (g/L)	11.67 ± 0.21	12.04 ± 0.05	ns
T-CHO (mmol/L)	11.35 ± 1.61	7.59 ± 0.48	*
TG (mmol/L)	15.54 ± 2.28	3.56 ± 0.04	***
HDL-C (mmol/L)	7.23 ± 1.07	3.06 ± 0.24	*
LDL-C (mmol/L)	6.11 ± 0.64	3.14 ± 0.08	**
GLU (mmol/L)	4.35 ± 0.24	6.68 ± 0.17	***
BA (μmol/L)	303.30 ± 24.61	246.15 ± 8.60	*
BUN (mmol/L)	3.2 ± 0.35	1.27 ± 0.13	**
ALP (U/L)	22.73 ± 4.65	11.79 ± 2.03	*
ALT (U/L)	2.52 ± 0.12	5.73 ± 1.43	*
AST (U/L)	7.10 ± 3.51	26.35 ± 1.53	**

Significant differences between the two groups are identified with different markers (* *p* < 0.05; ** *p* < 0.01; *** *p* < 0.001; ns, no significant difference). TP: total protein, ALB: albumin, T-CHO: total cholesterol, TG: triglyceride, HDL-C: high-density lipoprotein cholesterol, LDL-C: low-density lipoprotein cholesterol, GLU: glucose, BA: blood ammonia, BUN: blood urea nitrogen, ALP: alkaline phosphatase, ALT: alanine aminotransferase, AST: aspartate aminotransferase.

## Data Availability

The datasets generated and/or analyzed during the current study are not publicly available owing to security protocols and privacy regulations, but they may be made available on reasonable request to the corresponding author.

## Supplementary Materials

The following supporting information can be downloaded at: https://www.mdpi.com/article/10.3390/metabo14080432/s1, Figure S1: Experimental flow chart; Figure S2: Comparative analysis of intestine’s digestive enzymes (lipase, trypsin, amylase) and livers’ digestive genes (*lipase*, *trypsin*, *amylase*) between NGE and GRE; Figure S3: Photomicrograph of the mid-intestine of NGE (A) and GRE (B); Table S1: The primers sequences of qRT-PCR.

## References

[1] Lugert V., Thaller G., Tetens J., Schulz C., Krieter J. (2016). A review on fish growth calculation: Multiple functions in fish production and their specific application. Rev. Aquac..

[2] Wu L.A., Yang Y., Wang X., Weng Z.Y., Hua S.J., Li D., Xia J.H., Liu X.C., Meng Z.N. (2023). Genome-wide QTL mapping and RNA-seq reveal the genetic variation influencing growth traits in giant grouper (*Epinephelu lanceolatus*). Aquaculture.

[3] Guo J.R., Lin J.B., Li X.S., Wang L., Song K., Lu K.L., Zhang C.X. (2023). Enhanced intestinal microflora composition and phosphorus-transportation efficiency in fast-growing spotted seabass (*Lateolabrax maculatus*) fed a low-phosphorus diet. Aquaculture.

[4] Lin Z.J., Zhang Z.Y., Solberg M.F., Chen Z.Q., Wei M.L., Zhu F., Jia C.F., Meng Q., Zhang Z.W. (2021). Comparative transcriptome analysis of mixed tissues of black porgy (*Acanthopagrus schlegelii*) with differing growth rates. Aquac. Res..

[5] Chen B.L., Xiao W., Li D.Y., Zou Z.Y., Zhu J.L., Yu J., Yang H. (2024). Characterization of glucose metabolism in high-growth performance Nile tilapia (*Oreochromis niloticus*). Aquaculture.

[6] Meng K.F., Lin X., Chen Y.Y., Hu M.D., Hu W., Luo D.J. (2023). Integrated analysis of the digestive tract bacterial community on individual growth in sibling generation of Swamp Eels (*Monopterus albus*). Aquaculture.

[7] Goodrich H.R., Clark T.D. (2023). Why do some fish grow faster than others?. Fish Fish..

[8] Kestemont P., Jourdan S., Houbart M., M’elard C., Paspatis M., Fontaine P., Cuvier A., Kentouri M., Baras E. (2003). Size heterogeneity, cannibalism and competition in cultured predatory fish larvae: Biotic and abiotic influences. Aquaculture.

[9] Sherzada S., Sharif M.N., Ali Q., Khan S.A., Shah T.A., El-Tabakh M.A.M., Aziz T., Nabi G., Alharbi M., Albekairi T.H. (2023). Relative expression levels of growth hormone gene and growth rate in Indian major carp species. Acta Biochim. Pol..

[10] Marín A., Alonso A.M., Delgadin T.H., López-Landavery E.A., Cometivos L.J., Saavedra-Flores A., Reyes-Flores L.E., Yzásiga-Barrera C.G., Fernandino J.I., Zelada-Mázmela E. (2023). Analysis of truncated growth hormone receptor 1 in the differential growth of fine flounder *Paralichthys adspersus*. Aquaculture.

[11] Allen D., Rosenfeld J., Richards J. (2016). Physiological basis of metabolic trade-offs between growth and performance among different strains of rainbow trout. Can. J. Fish. Aquat. Sci..

[12] Li W.S., Lin H.R. (2010). The endocrine regulation network of growth hormone synthesis and secretion in fish: Emphasis on the signal integration in somatotropes. Sci. China Life Sci..

[13] Reindl K.M., Sheridan M.A. (2012). Peripheral regulation of the growth hormone-insulin-like growth factor system in fish and other vertebrates. Comp. Biochem. Physiol. A Mol. Integr. Physiol..

[14] Murua H., Rodriguez-Marin E., Neilson J.D., Farley J.H., Juan-Jordá M.J. (2017). Fast versus slow growing tuna species: Age, growth, and implications for population dynamics and fisheries management. Rev. Fish Biol. Fish..

[15] Sousa T., Domingos T., Poggiale J.C., Kooijman S. (2010). Dynamic energy budget theory restores coherence in biology. Philos. Trans. R. Soc. Lond. B Biol. Sci..

[16] Blanco A.M. (2020). Hypothalamic- and pituitary-derived growth and reproductive hormones and the control of energy balance in fish. Gen. Comp. Endocr..

[17] Canosa L.F., Bertucci J.I. (2020). Nutrient regulation of somatic growth in teleost fish. The interaction between somatic growth, feeding and metabolism. Mol. Cell. Endocrinol..

[18] Näslund E., Hellström P.M. (2007). Appetite signaling: From gut peptides and enteric nerves to brain. Physiol. Behav..

[19] Beullens K., Eding E.H., Ollevier F., Komen J., Richter C.J.J. (1997). Sex differentiation, changes in length, weight and eye size before and after metamorphosis of European eel (*Anguilla anguilla* L.) maintained in captivity. Aquaculture.

[20] Damusaru J.H., Moniruzzaman M., Park Y., Seong M., Jung J.Y., Kim D.J., Bai S.C. (2019). Evaluation of fish meal analogue as partial fish meal replacement in the diet of growing Japanese eel *Anguilla japonica*. Anim. Feed Sci. Technol..

[21] Tanaka H. (2015). Progression in artificial seedling production of Japanese eel *Anguilla japonica*. Fish. Sci..

[22] Degani G., Gallagher M.L. (2010). The relationship between growth, food conversion and oxygen consumption in developed and undeveloped American eels *Anguilla rostrata* (L.). J. Fish Biol..

[23] Aya F.A., Unida J.C.L., Garcia L.M.B. (2023). Effect of size grading on growth of yellow Pacific shortfin eel (*Anguilla bicolor pacifica*). J. Fish Biol..

[24] Zhai S.W., Zhao P.Y., Huang L.X. (2020). Dietary bile acids supplementation improves the growth performance with regulation of serum biochemical parameters and intestinal microbiota of growth retarded European eels (*Anguilla anguilla*) cultured in cement tanks. Isr. J. Aquacult-Bamid..

[25] Zhai S.W., Zhao P.Y., Shi Y., Chen X.H., Liang Y. (2018). Effects of Dietary Surfactin Supplementation on Growth Performance, Intestinal Digestive Enzymes Activities, and Hepatic Antioxidant Potential of American Eel (*Anguilla rostrata*) Elvers. Isr. J. Aquacult-Bamid..

[26] Zhai S.W., Shi Q.C., Chen X.H. (2016). Effects of Dietary Surfactin Supplementation on Growth, Digestive Enzyme Activity, and Antioxidant Potential in the Intestine of Growth Retarded Marbled Eel (*Anguilla marmaorata*) at Elver Stage. Isr. J. Aquacult-Bamid..

[27] Lin M., Zeng C.X., Jia X.Q., Zhai S.W., Li Z.Q., Ma Y. (2019). The composition and structure of the intestinal microflora of *Anguilla marmorata* at different growth rates: A deep sequencing study. J. Appl. Microbiol..

[28] Willemse J.J. (1976). Characteristics of myotomal muscle fibres and their possible relation to growth rate in eels—*Anguilla anguilla* (L.) (*Pisces, Teleostei*). Aquaculture.

[29] Triantaphyllopoulos K.A., Cartas D., Miliou H. (2020). Factors influencing GH and IGF-I gene expression on growth in teleost fish: How can aquaculture industry benefit?. Rev. Aquac..

[30] Kaneko N., Ishikawa T., Nomura K. (2023). Effects of the short-term fasting and refeeding on growth-related genes in Japanese eel (*Anguilla japonica*) larvae. Comp. Biochem. Physiol. B Biochem. Mol. Biol..

[31] Yang B.Y., Green M., Chen T.T. (1999). Early embryonic expression of the growth hormone family protein genes in the developing rainbow trout, *Oncorhynchus mykiss*. Mol. Reprod. Dev..

[32] Sudo R., Kawakami Y., Nomura K., Tanaka H., Kazeto Y. (2022). Production of recombinant Japanese eel (*Anguilla japonica*) growth hormones and their effects on early-stage larvae. Gen. Comp. Endocrinol..

[33] Zhong H., Xiao J., Chen W.Z., Zhou Y., Tang Z.Y., Guo Z.B., Luo Y.J., Lin Z.B., Gan X., Zhang M. (2014). DNA methylation of pituitary growth hormone is involved in male growth superiority of Nile tilapia (*Oreochromis niloticus*). Comp. Biochem. Physiol. B Biochem. Mol. Biol..

[34] Peres H., Santos S., Oliva-Teles A. (2014). Blood chemistry profile as indicator of nutritional status in European seabass (*Dicentrarchus labrax*). Fish Physiol. Biochem..

[35] Dagoudo M., Qiang J., Bao J.W., Tao Y.F., Zhu T.H.J., Tumukunde E.M., Ngoepe T.K., Xu P. (2021). Effects of acute hypoxia stress on hemato-biochemical parameters, oxidative resistance ability, and immune responses of hybrid yellow catfish (*pelteobagrus fulvidraco × P. vachelli*) juveniles. Aquac. Int..

[36] Chen B.L., Xiao W., Zou Z.Y., Zhu J.L., Li D.Y., Yu J., Yang H. (2022). Comparing Transcriptomes Reveals Key Metabolic Mechanisms in Superior Growth Performance Nile Tilapia (*Oreochromis niloticus*). Front. Genet..

[37] Wang Z., Qian X.Q., Xie S.Q., Yun B. (2020). Changes of growth performance and plasma biochemical parameters of hybrid grouper (*Epinephelus lanceolatus♂ x Epinephelus fuscoguttatus♀*) in response to substitution of dietary fishmeal with poultry by-product meal. Aqua. Rep..

[38] Yin X.L., Li Z.J., Yang K., Lin H.Z., Guo Z.X. (2014). Effect of guava leaves on growth and the non-specific immune response of *Penaeus monodon*. Fish Shellfish Immunol..

[39] Li Q.Z., Wang J., Chen Y.Y., Wu X.Y., Liu Y., Lai J.S., Song M.J., Li F.Y., Li P.C., He B. (2024). Comparison of muscle structure and transcriptome analysis reveals the mechanism of growth variation in Yangtze sturgeon (*Acipenser dabryanus*). Aquaculture.

[40] Zhang B., Jiang D., Zhang D.L., Wang Z.Y., Fang M. (2023). Comparative analysis of transcriptome of muscle tissue of individuals with different growth rate of *Larimichthys crocea*. J. Fish. China.

[41] Zhang W., Liu K., Tan B.P., Liu H.Y., Dong X.H., Yang Q.H., Chi S.Y., Zhang S., Wang H.L. (2019). Transcriptome, enzyme activity and histopathology analysis reveal the effects of dietary carbohydrate on glycometabolism in juvenile largemouth bass, *Micropterus salmoides*. Aquaculture.

[42] Feng Q.M. (2023). Study on Behavioral and Physiological Mechanism of Individual Growth Differences of *Apostichopus japonicus*. Ph.D. Thesis.

[43] Li C., Sun L.D., Lin H.Z., Qin Z.D., Tu J.G., Li J., Chen K.P., Babu V.S., Lin L. (2019). Glutamine starvation inhibits snakehead vesiculovirus replication via inducing autophagy associated with the disturbance of endogenous glutathione pool. Fish Shellfish Immunol..

[44] Sakyi M.E., Cai J., Ampofo-Yeboah A., Anokyewaa M.A., Wang Z.W., Jian J.C. (2021). Starvation and re-feeding influence the growth, immune response, and intestinal microbiota of Nile tilapia (*Oreochromis niloticus; Linnaeus 1758*). Aquaculture.

[45] Zhao J., Feng L., Liu Y., Jiang W.D., Wu P., Jiang J., Zhang Y.G., Zhou X.Q. (2014). Effect of dietary isoleucine on the immunity, antioxidant status, tight junctions and microflora in the intestine of juvenile Jian carp (*Cyprinus carpio var. Jian*). Fish Shellfish Immunol..

[46] Magnoni L.J., Novais S.C., Eding E., Leguen I., Lemos M.F.L., Ozório R.O.A., Geurden I., Prunet P., Schrama J.W. (2019). Acute Stress and an Electrolyte-Imbalanced Diet, but Not Chronic Hypoxia, Increase Oxidative Stress and Hamper Innate Immune Status in a Rainbow Trout (*Oncorhynchus mykiss*) Isogenic Line. Front. Physiol..

[47] Wu X., Lai J., Chen Y., Liu Y., Song M., Li F., Li P., Li Q., Gong Q. (2023). Combination of metabolome and proteome analyses provides insights into the mechanism underlying growth differences in *Acipenser dabryanus*. iScience.

[48] Ronnestad I., Gomes A.S., Murashita K., Angotzi R., Jönsson E., Volkoff H. (2017). Appetite-Controlling Endocrine Systems in Teleosts. Front. Endocrinol..

[49] Li S.S., Zhao L.P., Xiao L., Liu Q.Y., Zhou W.Y., Qi X., Chen H.P., Yang H.R., Liu X.C., Zhang Y. (2012). Structural and functional characterization of neuropeptide Y in a primitive teleost, the Japanese eel (*Anguilla japonica*). Gen. Comp. Endocrinol..

[50] Alrubaian J., Lecaude S., Barba J., Szynskie L., Jacobs N., Bauer D., Brown C., Kaminer I., Bagrosky B., Dores R.M. (2006). Trends in the evolution of the prodynorphin gene in teleosts: Cloning of eel and tilapia prodynorphin cDNAs. Peptides.

[51] Kurokawa T., Iinuma N., Unuma T., Tanaka H., Kagawa H., Ohta H., Suzuki T. (2004). Development of endocrine system regulating exocrine pancreas and estimation of feeding and digestive ability in Japanese eel larvae. Aquaculture.

[52] Yada T., Abe M., Kaifu K., Yokouchi K., Fukuda N., Kodama S., Hakoyama H., Ogoshi M., Kaiya H., Sakamoto T. (2020). Ghrelin and food acquisition in wild and cultured Japanese eel (*Anguilla japonica*). Comp. Biochem. Physiol. A Mol. Integr. Physiol..

[53] Cerdá-Reverter J.M., Sorbera L.A., Carrillo M., Zanuy S. (1999). Energetic dependence of NPY-induced LH secretion in a teleost fish (*Dicentrarchus labrax*). Am. J. Physiol..

[54] Silverstein J.T., Breininger J., Baskin D.G., Plisetskaya E.M. (1998). Neuropeptide Y-like gene expression in the salmon brain increases with fasting. Gen. Comp. Endocrinol..

[55] Narnaware Y.K., Peter R.E. (2001). Effects of food deprivation and refeeding on neuropeptide Y (NPY) mRNA levels in goldfish. Comp. Biochem. Physiol. B Biochem. Mol. Biol..

[56] Zhang Y.F., Gao Y.F., Wang J.R., Yu X.M., Zhang Z., Tong J.G. (2023). Expression analyses of npy and pomc genes in extreme growthand starvation-refeeding bighead carp (*hypophthalmichthys nobilis*). Acta Hydrobiol. Sin..

[57] Huang L.L., Tan H.Y., Fogarty M.J., Andrews Z.B., Veldhuis J.D., Herzog H., Steyn F.J., Chen C. (2014). Actions of NPY, and Its Y1 and Y2 Receptors on Pulsatile Growth Hormone Secretion during the Fed and Fasted State. J. Neurosci..

[58] Kiris G.A., Kumlu M., Dikel S. (2007). Stimulatory effects of neuropeptide Y on food intake and growth of *Oreochromis niloticus*. Aquaculture.

[59] Breton B., Mikolajczyk T., Popek W., Bieniarz K., Epler P. (1991). Neuropeptide Y stimulates in vivo gonadotropin secretion in teleost fish. Gen. Comp. Endocrinol..

[60] Tine M., Kuhl H., Teske P.R., Tschöp M.H., Jastroch M. (2016). Diversification and coevolution of the ghrelin/growth hormone secretagogue receptor system in vertebrates. Ecol. Evol..

[61] Riley L.G., Fox B.K., Kaiya H., Hirano T., Grau E.G. (2005). Long-term treatment of ghrelin stimulates feeding, fat deposition, and alters the GH/IGF-I axis in the tilapia, *Oreochromis mossambicus*. Gen. Comp. Endocrinol..

[62] Yuan X.C., Cai W.J., Liang X.F., Su H., Yuan Y.C., Li A.X., Tao Y.X. (2015). Obestatin partially suppresses ghrelin stimulation of appetite in “high-responders” grass carp, *Ctenopharyngodon idellus*. Comp. Biochem. Physiol. A Mol. Integr. Physiol..

[63] Tinoco A.B., Näslund J., Delgado M.J., de Pedro N., Johnsson J.I., Jönsson E. (2014). Ghrelin increases food intake, swimming activity and growth in juvenile brown trout (*Salmo trutta*). Physiol. Behav..

[64] Miura T., Maruyama K., Shimakura S.I., Kaiya H., Uchiyama M., Kangawa K., Shioda S., Matsuda K. (2007). Regulation of food intake in the goldfish by interaction between ghrelin and orexin. Peptides.

[65] Matsuda K. (2013). Regulation of feeding behavior and psychomotor activity by corticotropin-releasing hormone (CRH) in fish. Front. Neurosci..

[66] Wang T., Zhou C.W., Yuan D.Y., Lin F.J., Chen H., Wu H.W., Wei R.B., Xin Z.M., Liu J., Gao Y.D. (2014). *Schizothorax prenanti* corticotropin-releasing hormone (CRH): Molecular cloning, tissue expression, and the function of feeding regulation. Fish Physiol. Biochem..

[67] Bauer P.V., Hamr S.C., Duca F.A. (2016). Regulation of energy balance by a gut-brain axis and involvement of the gut microbiota. Cell. Mol. Life Sci..

[68] Volkoff H. (2014). Appetite regulating peptides in red-bellied piranha, *Pygocentrus nattereri*: Cloning, tissue distribution and effect of fasting on mRNA expression levels. Peptides.

[69] Chen Y., Pandit N.P., Fu J.J., Li D., Li J.L. (2014). Identification, characterization and feeding response of peptide YYb (PYYb) gene in grass carp (*Ctenopharyngodon idellus*). Fish Physiol. Biochem..

