# Supplementary material for: Characterizing Growth-Retarded Japanese Eels (Anguilla japonica): Insights into Metabolic and Appetite Regulation
Source: Metabolites. 2024 Aug 5;14(8):432. doi: 10.3390/metabo14080432 (PMC11356357; doi:10.3390/metabo14080432)
Supplement: Supplementary file 1 [file metabolites-14-00432-s001.zip › Figure S2 .pdf]

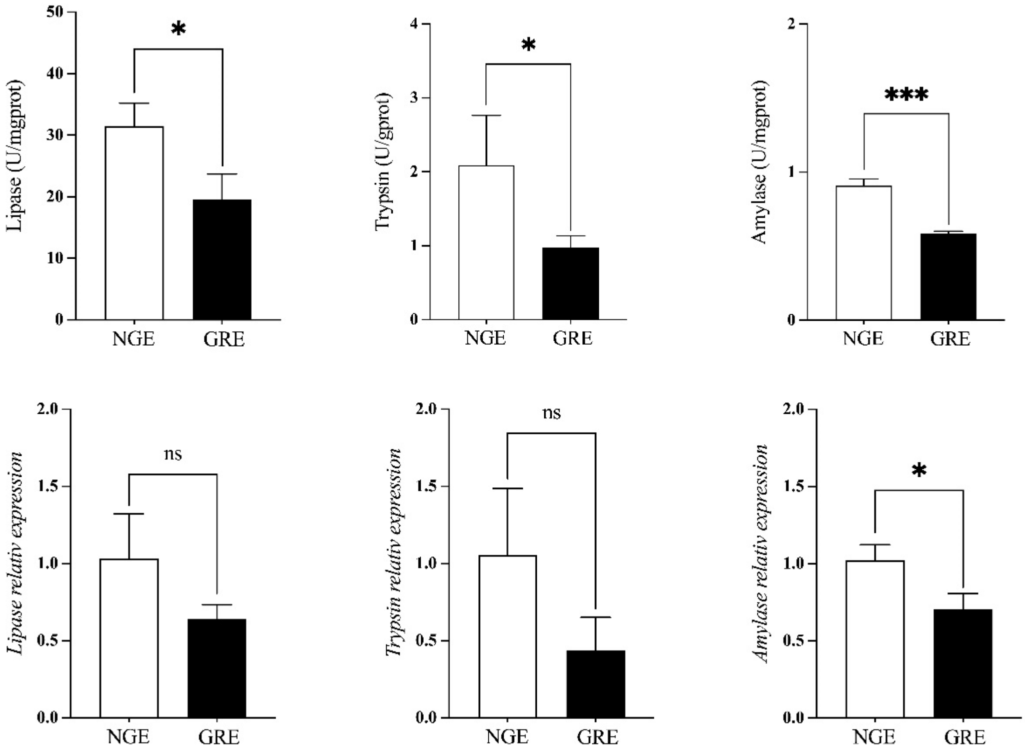

Figure S2. Comparative analysis of intestine's digestive enzymes (lipase, trypsin, amylase) and livers' digestive genes (*lipase*, *trypsin*, *amylase*) between NGE and GRE. Significant differences between the two groups are identified with different markers (* $p < 0.05$; *** $p < 0.001$; ns, no significant difference).
