# Supplementary material for: Characterizing Growth-Retarded Japanese Eels (Anguilla japonica): Insights into Metabolic and Appetite Regulation
Source: Metabolites. 2024 Aug 5;14(8):432. doi: 10.3390/metabo14080432 (PMC11356357; doi:10.3390/metabo14080432)
Supplement: Supplementary file 1 [file metabolites-14-00432-s001.zip › Figure S3.pdf]

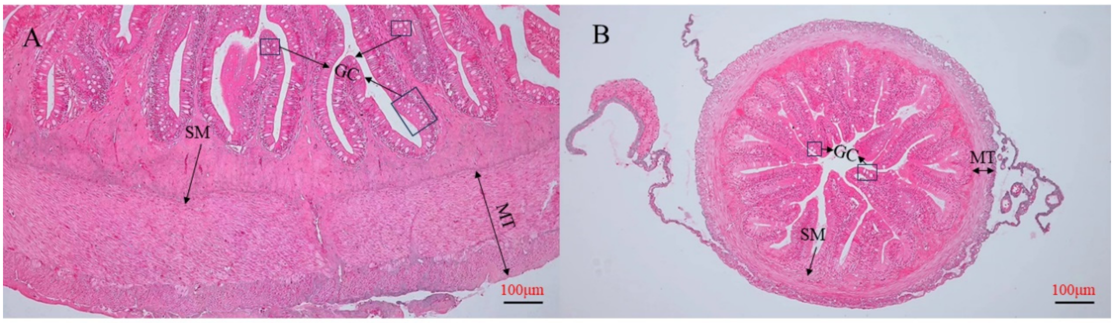

Figure S3. Photomicrograph of the mid-intestine of NGE (A) and GRE (B). MT, muscular thickness; SM, submucosa; GC, goblet cells (bright or transparent).
