# Supplementary material for: Characterizing Growth-Retarded Japanese Eels (Anguilla japonica): Insights into Metabolic and Appetite Regulation
Source: Metabolites. 2024 Aug 5;14(8):432. doi: 10.3390/metabo14080432 (PMC11356357; doi:10.3390/metabo14080432)
Supplement: Supplementary file 1 [file metabolites-14-00432-s001.zip › Table S1 .pdf]

Table S1. The primers sequences of qRT-PCR

	Gene	Forward primer (5′-3′)	Reverse primer (5′-3′)	Accession number
Appetite				
	npy	GGAAGGATACCCGTCCAAAC	CAGCAGAAGCTCTGTAAGCAG	JX070034
	pomc	TTCCTGCTCCCTATGGTGT	GCTGTCCTTCTTGGTCTGG	AY158010.1
	crh	CAACCGTAAGTGGGAGAGGA	GATCAGACTGTGGACCAGGA	LC010940.1
	cck	AACCCAAAGCAGAGGAGG	GCTCGCTCGGCTGTTTAT	AB109556
	pyy	ATGGCCGTAGTGCTGAAACCCTGGA	TCACCACATGTAGGAGTCGTCGTA	AB109557
	ghrelin	AAACGCACCGCATACATCAT	TGGACCAGCTCCTCAGTCAT	AB062427
Growth				
	gh	AACGCAGGAGAAATCGGATG	GCTGTTTGAGAAAGCATCGC	M24066
	ghr1	GGCAACATCACCCCTTAATC	GTGTGTTGCGCTGGTAAATC	AB180476
	igf1	CTTGTGCCCAAGACAAAACC	TGCAGATCGATCAACTCCAG	AB180476
Digestion				
	amylase	GTGGAGAACCCATTACGGCA	CCATCCTTCACCCCAGGTTC	AB070721.1
	lipase	CCGAGCTTCCAAGGTTCTCC	GGGTTCATAGTGTTGCCCCA	AB519643.1
	trypsin	GGGTTTGCTGGGAGCTACTT	GCGTCCACAAATATGGCGTC	EU715406.1
	ef1α	TGTGGGAGTCAACAAGATGGA	CTCAAAACGCTTCTGGCTGTA	EU407824
