# Supplementary figures and images for: Characterizing Growth-Retarded Japanese Eels (Anguilla japonica): Insights into Metabolic and Appetite Regulation
Source: Metabolites. 2024 Aug 5;14(8):432. doi: 10.3390/metabo14080432 (PMC11356357; doi:10.3390/metabo14080432)

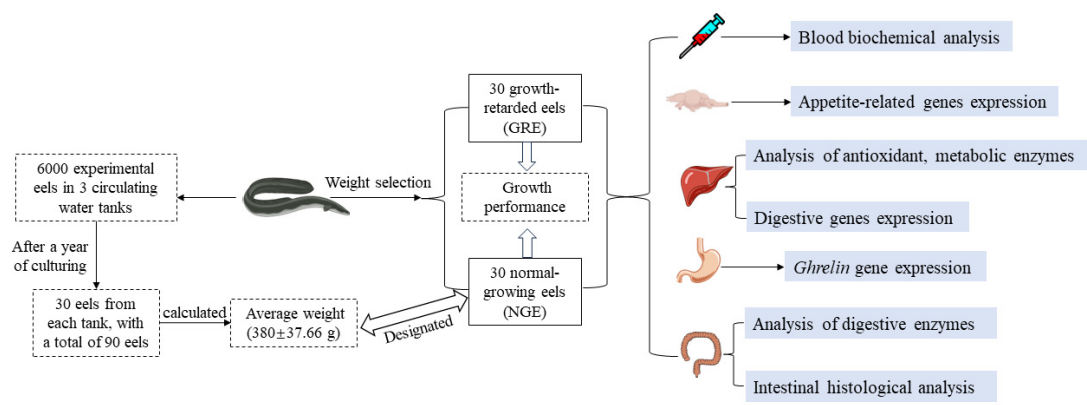

**Figure S1.** Experimental flow chart.

Supplement: Supplementary file 1 [file metabolites-14-00432-s001.zip › Figure S1.pdf]
